# Balloon internal fixation: A novel approach to manipulate catheter knotting

**DOI:** 10.1002/ccr3.4981

**Published:** 2021-11-12

**Authors:** Xian‐Liang Yan, Yu‐Tong Cheng, Tao Sun

**Affiliations:** ^1^ Division of Cardiology Beijing Anzhen Hospital Beijing Institute of Heart, Lung, and Blood Vessel Diseases Capital Medical University Beijing China

**Keywords:** balloon internal fixation, catheter knotting, trans‐radial access

## Abstract

A successful alternative technique to resolve the catheter knotting during radial access using balloon internal fixation of 5F angiographic catheter in the cath laboratory.

## Graphical abstract

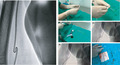

## 1 INTRODUCTION

Trans‐radial approach currently is a more common approach during percutaneous coronary angiography and intervention. Catheter knotting is still a matter of concern for an interventional cardiologist. Hereby, we describe a successful alternative technique to resolve the catheter knotting during radial access using balloon internal fixation of 5F angiographic catheter in the cath laboratory.

Trans‐radial access (TRA) has become the first choice for coronary intervention, with few complications and more comfortable for patients.^1^, ^2^ However, this novel access may lead to severe catheter twisting and following manipulation may be required to unravel the catheter and to avoid complications.^3^ Re‐rotate catheter can handle minor problems, but there were also some cases in which knot was so severe that cannot be handled by this method but for surgical intervention. In this report, we describe our technique to handle catheter knotting and report its short‐term outcome.

## 2 CASE PRESENTATION

**FIGURE 1:**
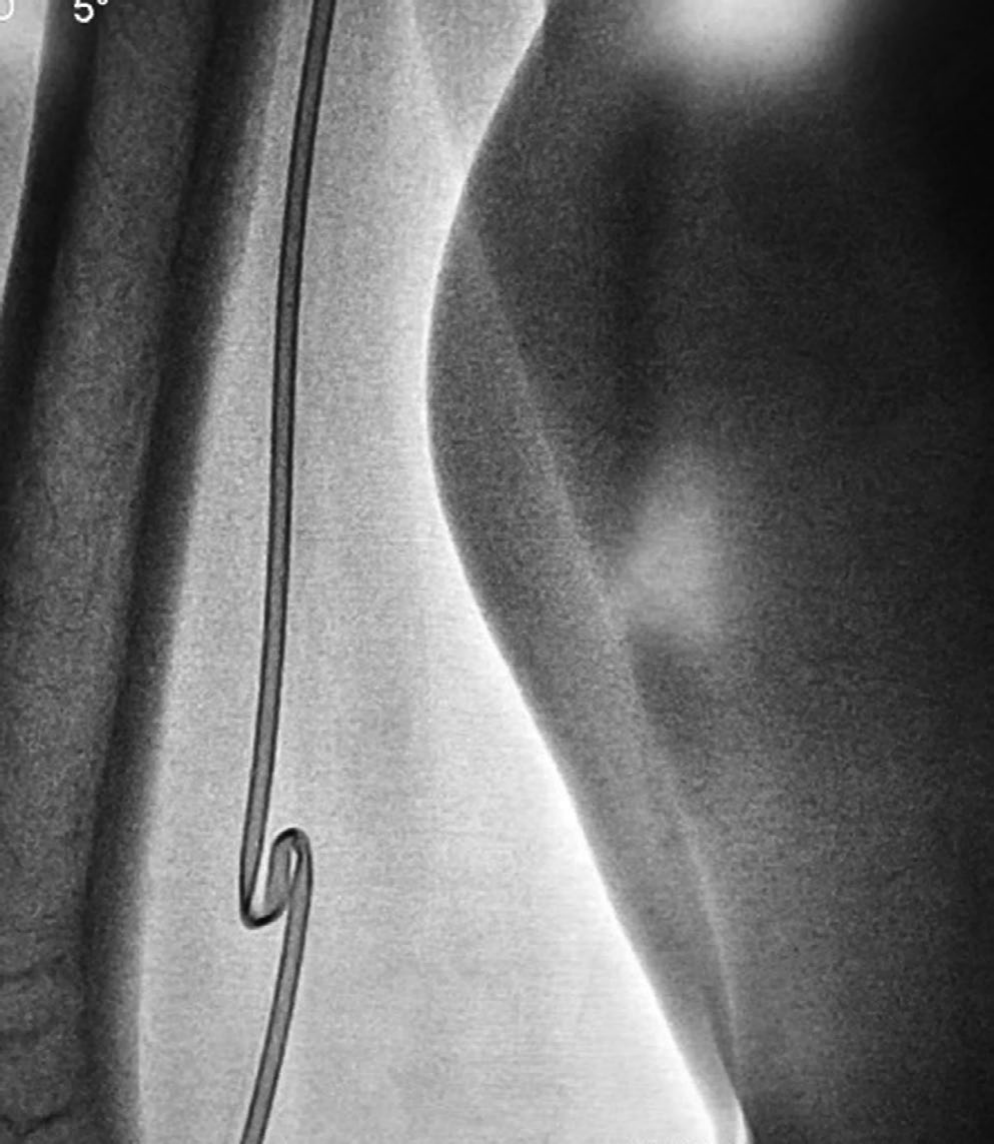
Revealed an outright knot within right brachial artery with fluoroscopy

**FIGURE 2:**
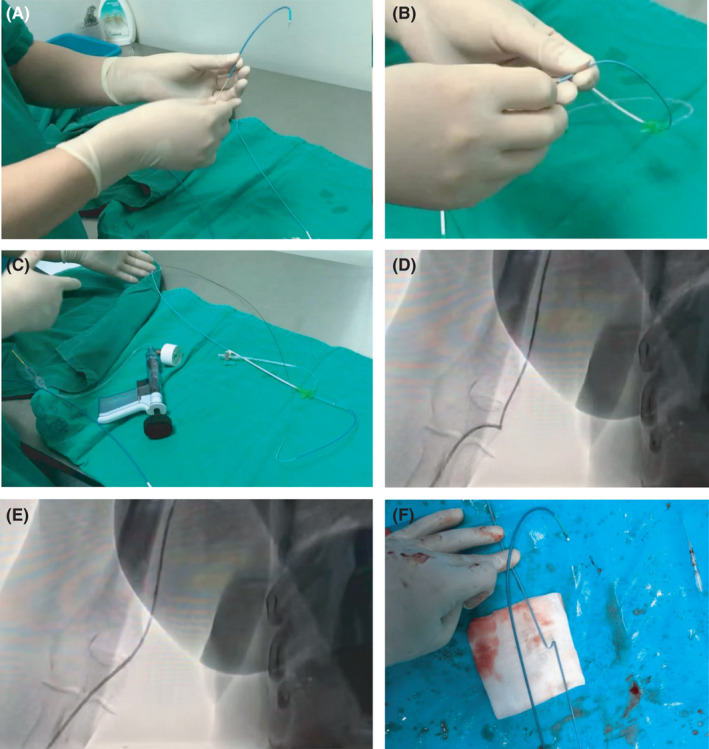
(A) Balloon passed through the cut guiding catheter from tail to tip. (B) A balloon passed through the cut angiographic catheter. (C) The balloon and the angiographic catheter contact tightly. (D) Under fluoroscopy, the balloon was carefully pulled while EBU 3.5 guiding catheter was pushed in. (E) Under fluoroscopy, the knot was untwisted successfully. (F) The knotted catheter was pulled out successfully

A 62‐year‐old woman with history of hypertension underwent selective coronary angiography because of intermittent chest pain for 10 days. The right radial artery was the access of choice. After easy cannulation, 6 French transradial sheath that was Radifocus introducer II M Coat Hydrophilic Radial Access introducer kit (Terumo Medical Corporation) was advanced and 5 French multifunctional angiographic catheter (Terumo Medical Corporation) was introduced into aortic sinus. Initially, subclavian tortuous anatomy made catheter rotation redundancy, and then, the pressure curve was partial dampening, and with fluoroscopy, it revealed an outright knot within right brachial artery (Figure 1). Gentle traction or rotation did not allow catheter withdrawal. As any vasodilator could not be used to treat radial artery spasm locally, we decided to untwist the catheter knot by balloon internal fixation and the details were as follows: Cutoff the tail of discounted catheter and the tail of 6 French EBU3.5 guiding catheter (Medtronic). A 2.0 × 20 mm balloon (Sprinter legend, Medtronic) passed through the cut guiding catheter from tail to tip (Figure 2A) and then passed through the cut angiographic catheter (Figure 2B). When the balloon all passed through the cut angiographic catheter, the balloon was given atmospheric pressure of about 14 atm and makes sure that the balloon and the angiographic catheter contact tightly (Figure 2C). The guiding catheter was pushed to the knot point along angiographic catheter by left hand. The balloon was pulled out gently under fluoroscopy while guiding catheter was pushed in (2D), and the knotted catheter was pulled out successfully (Figure 2E and Figure 2F). During the whole traction period, there was no discomfort of patient and the tip of angiographic catheter was complete. Contrast was injected from the sheath to confirm patency of the brachial artery. After 10 days’ follow‐up, there was no discomfort in right upper limb of the patient.

## 3 DISCUSSION

There are many reasons for angiographic catheter knotting, including vascular tortuosity, vasospasm, abnormal ostial of coronary artery, unskilled manipulation, excessive traction, and rotation. Understanding the tortuosity of blood vessels in advance, and avoiding excessive rotation of the catheter, maintaining a guide wire in the catheter lumen can help us to prevent catheter from looping.

Once the catheter was knotted, inadequate manipulation and excessive traction can make catheter fracture or even artery dissection.^4^, ^5^ There were several methods to retrieve catheter knot, such as gentle rotation of the catheter to the opposite direction, advancement of a super stiff wire into the loop, external fixation of the distal part of the catheter at the arm level with a blood pressure cuff,^6^ snare delivery.^7^, ^8^, ^9^ In catheter ablation procedure, steerable sheath was also used to untie diagnostic decapolar catheter knot.^10^

When it was difficult to withdraw the knotted catheter, we can advance a bigger cut guiding catheter outside the angiographic catheter to strengthen the supporting power and exchange the shape of angiographic catheter. Pushing the guiding catheter into the knot point and pulling the angiographic catheter gently at the same time can help us to withdraw the knotted catheter. In our case, the internal diameter of 6 French EBU guiding catheter was 1.8mm, which was bigger than the external diameter of 5 French angiographic catheter, which was 1.7 mm, and makes sure that the guiding catheter can enclose the angiographic catheter. According to Aminian's study,^11^ the length of guiding catheter was between “Knotted point‐ Puncture point” distance and “Knotted point‐ Cut point” distance, and the length of exceeded‐guiding catheter part of angiographic catheter was too short to fix, so that we make balloon and angiographic catheter contact tightly, and as a result, we prolong the length of exceeded‐guiding catheter part of angiographic catheter sufficiently in order to facilitate the procedure, which was a bright point of our case.

## 4 CONCLUSION

The method of balloon internal fixation of 5F angiographic catheter was simple, practical, and economical, and this method can reduce artery injury also can avoid surgery. It provided us with a new method and choice to handle catheter knotting.

## CONFLICTS OF INTEREST

We report no competing interest associated with the work reported in this manuscript.

## AUTHOR CONTRIBUTIONS

Yu‐Tong Cheng contributed to the discussion and edited the manuscript. Tao Sun participated in the data collection. Xian‐Liang Yan wrote the manuscript. All authors read and approved the final manuscript.

## CONSENT

Written informed consent was obtained from the patient to publish this case report.

## Data Availability

All data included in this study are available upon request by contact with the corresponding author.
